# Additive effects of blood glucose lowering drugs, statins and renin-angiotensin system blockers on all-site cancer risk in patients with type 2 diabetes

**DOI:** 10.1186/1741-7015-12-76

**Published:** 2014-05-13

**Authors:** Alice PS Kong, Xilin Yang, Wing-Yee So, Andrea Luk, Ronald CW Ma, Risa Ozaki, Kitty KT Cheung, Heung-Man Lee, Linda Yu, Gang Xu, Chun-Chung Chow, Juliana CN Chan

**Affiliations:** 1 Department of Medicine and Therapeutics, The Chinese University of Hong Kong, Prince of Wales Hospital, Shatin, Hong Kong, SAR, China; 2 Department of Epidemiology and Biostatistics, School of Public Health, Tianjin Medical University, Tianjin 300070, China; 3 Hong Kong Institute of Diabetes and Obesity, Shatin, Hong Kong, SAR, China; 4 Li Ka Shing Institute of Health Sciences, The Chinese University of Hong Kong, Prince of Wales Hospital, Shatin, Hong Kong, SAR, China; 5 Hong Kong Hospital Authority Headquarter, Hong Kong, SAR, China

**Keywords:** Cancer risk, Type 2 diabetes, Glycemic control, Statins, Renin-angiotensin-system inhibitors

## Abstract

**Background:**

Hyperglycemia is associated with increased risk of all-site cancer that may be mediated through activation of the renin-angiotensin-system (RAS) and 3-hydroxy-3-methyl-glutaryl-coenzyme-A-reductase (HMGCR) pathways. We examined the joint associations of optimal glycemic control (HbA_1c_ <7%), RAS inhibitors and HMGCR inhibitors on cancer incidence in patients with type 2 diabetes.

**Methods:**

Patients with type 2 diabetes, with or without a history of cancer or prior exposure to RAS or HMGCR inhibitors at baseline were observed between 1996 and 2005. All patients underwent a comprehensive assessment at baseline and were followed until the censored date at 2005 or their death.

**Results:**

After a median follow-up period of 4.91 years (interquartile range, 2.81 to 6.98), 271 out of 6,103 patients developed all-site cancer. At baseline, patients with incident cancers were older, had longer disease duration of diabetes, higher alcohol and tobacco use, and higher systolic blood pressure and albuminuria, but lower triglyceride levels and estimated glomerular filtration rate (*P* <0.05). Patients who developed cancers during follow-up were less likely to have started using statins (22.5% versus 38.6%, *P* <0.001), fibrates (5.9% versus 10.2%, *P* = 0.02), metformin (63.8% versus 74.5%, *P* <0.001) or thiazolidinedione (0.7% versus 6.8%, *P* <0.001) than those who remained cancer-free. After adjusting for co-variables, new treatment with metformin (hazard ratio: 0.39; 95% confidence interval: 0.25, 0.61; *P* <0.001), thiazolidinedione (0.18; 0.04, 0.72; *P* = 0.015), sulphonylurea (0.44; 0.27, 0.73; *P* = 0.014), insulin (0.58; 0.38, 0.89; *P* = 0.01), statins (0.47; 0.31, 0.70; *P* <0.001) and RAS inhibitors (0.55; 0.39, 0.78; *P* <0.001) were associated with reduced cancer risk. Patients with all three risk factors of HbA_1c_ ≥7%, non-use of RAS inhibitors and non-use of statins had four-fold adjusted higher risk of cancer than those without any risk factors (incidence per 1,000-person-years for no risk factors: 3.40 (0.07, 6.72); one risk factor: 6.34 (4.19, 8.50); two risk factors: 8.40 (6.60, 10.20); three risk factors: 13.08 (9.82, 16.34); *P* <0.001).

**Conclusions:**

Hyperglycemia may promote cancer growth that can be attenuated by optimal glycemic control and inhibition of the RAS and HMGCR pathways.

## Background

Diabetes increases the risk of vascular, cancer, non-vascular and non-cancer deaths by 1.3 to 3-fold compared with non-diabetic subjects [1]. Our group [2] and others [1] have reported linear associations between glycemia (glycated hemoglobin (HbA_1c_) and fasting plasma glucose) and cancer risk in diabetic and non-diabetic subjects. Experimentally, chronic hyperglycemia can activate interlinked molecular pathways, resulting in oxidative stress, inflammation and abnormal cell cycles, which can be implicated in carcinogenesis [3-5]. In diabetes, while control of blood lipids, blood pressure, blood glucose and inhibition of the renin-angiotensin system (RAS) can reduce cardiovascular and/or renal events, cancer is emerging as an important co-morbidity, especially in areas such as Asia with lower prevalence of coronary heart disease compared to the West [6,7].

The Hong Kong Diabetes Registry was established in 1995, comprising a prospective cohort with documentation of risk factors, clinical outcomes and drug usage [8,9]. Using this Registry, we have made important observations: all-site cancer accounted for 25% of diabetes-related deaths, led by hepatocellular and colorectal cancer; a 1% increase in HbA_1c_ was associated with an 18% increased hazard ratio (HR) of all-site cancer after adjustment for confounders including drug use; use of insulin, sulphonylurea, metformin, thiazolidinedione (TZD), RAS inhibitors or statins was associated with reduced cancer risk; and there were non-linear risk associations between lipids and cancer [2].

**Figure 1:**
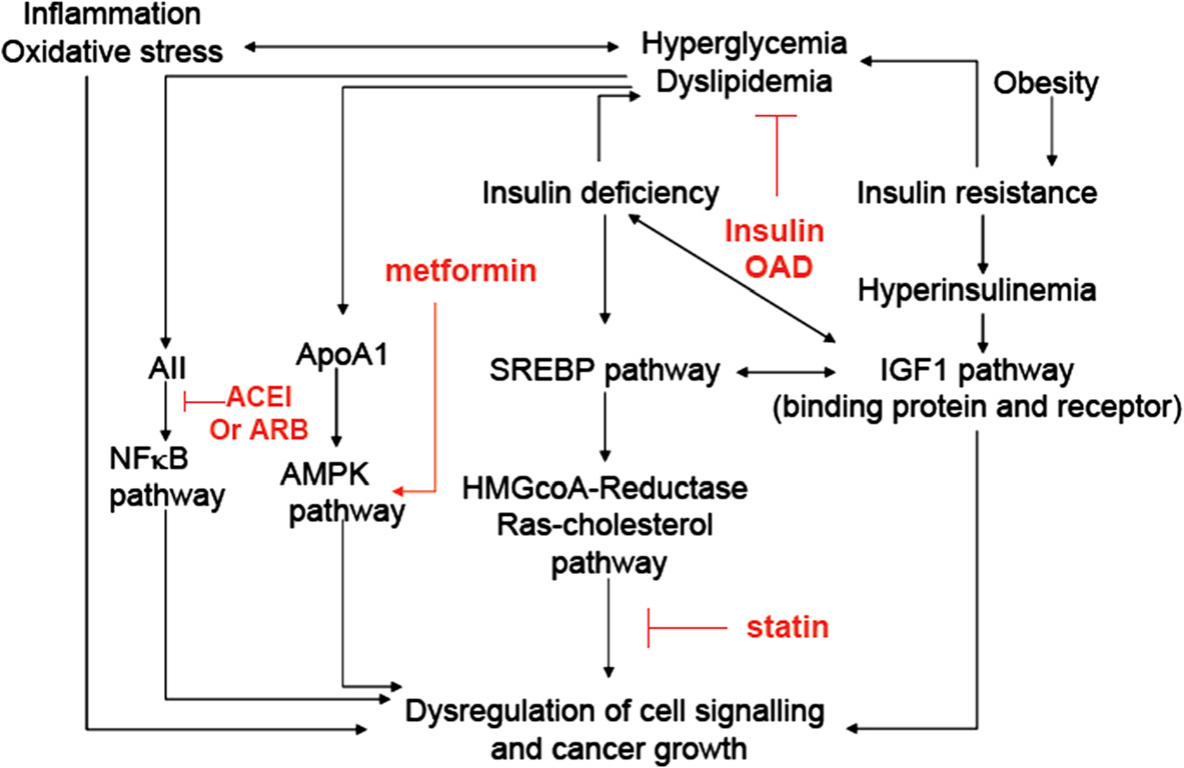
**A schematic diagram summarizing possible mechanisms underlying the risk associations of cancer with diabetes and the attenuating effects of statins, renin-angiotensin-system inhibitors (angiotensin-converting enzyme inhibitors and angiotensin II receptor blockers), insulin and oral anti-diabetic drugs on cancer risk.** Apart from obesity-associated insulin resistance, which can activate cell-signaling pathways such as the insulin-like growth factor-1 (IGF1) pathway to increase risk of cancer, hyperglycemia and dyslipidemia due to insufficient insulin action can activate angiotensin II, inflammatory and redox pathways that share multiple intracellular signals, including sterol regulatory element-binding proteins (SREBP), 3-hydroxy-3-methyl-glutaryl-CoA-reductase (HMGCR), adenosine monophosphate-activated protein kinase (AMPK) and nuclear factor kappa B (NF-KB) pathways to cause abnormal cell cycles to increase cancer risk. Treatment with RAS and HMGCR inhibitors together with correction of hyperglycemia including insulin, insulin sensitizers and insulin secretagogues can block these pathways at multiple sites to break the vicious cycles. ACEI, angiotensin-converting enzyme inhibitors; ARB, angiotensin II blockers; OAD, oral anti-diabetic drugs.

Based on these findings, we asked whether optimal glycemic control and the use of statins and RAS inhibitors could reduce risk of cancer in a synergistic manner due to the interlinking nature of these molecular pathways (Figure 1). We tested this hypothesis using the Hong Kong Diabetes Registry comprising a prospective cohort of 6,103 patients with type 2 diabetes without cancer or exposure to these drugs before enrolment.

## Methods

### Patients

The Hong Kong Diabetes Registry was established in 1995 as a quality improvement program, with a weekly enrolment of 30 to 50 ambulatory patients with diabetes who were referred from community- and hospital-based clinics. All patients underwent a comprehensive assessment of complications and risk factors and would be followed until their death. From 1 December 1996 to 9 January 2005, 7,387 patients with diabetes were enrolled in the Registry. After excluding 323 patients with type 1 diabetes (acute presentation with diabetic ketoacidosis, heavy ketonuria (urine value classed above 3+) or continuous requirement of insulin within one year of diagnosis), 5 with missing type of diabetes, 45 with non-Chinese or unknown ethnicity, 175 with a history of cancer or receiving anti-cancer treatment and 736 with missing values in any of the variables used in the analysis, 6,103 patients were included in this analysis. The study was approved by the Chinese University of Hong Kong Clinical Research Ethics Committee with written informed consent from all participants.

### Baseline assessment and endpoint ascertainment

The assessment method had been described previously [8,9]. After an 8-hour overnight fast, structured assessment of the eye, feet, urine and blood was performed on all enrolled patients. The eye examination included visual acuity, fundoscopy through dilated pupils or retinal photography. A Doppler ultrasound scan, monofilament and graduated tuning fork were used to examine the foot. Blood samples were taken for measurement of fasting plasma glucose, HbA_1c_, lipid profile (high-density lipoprotein cholesterol (HDL-C), low-density lipoprotein cholesterol (LDL-C) and triglyceride) and renal function. A sterile spot urine sample was used to measure albumin-to-creatinine ratio (ACR). We used the Chinese-calibrated Modification of Diet in Renal Disease study formula to estimate glomerular filtration rate (eGFR), expressed in ml/min/1.73 m^2^:

$$\begin{array}{c} \mathrm{eGFR}=186\times {\left[\mathrm{serum}\;\mathrm{creatinine}\times 0.011\right]}^{-1.154}\\ \;\times {\left[\mathrm{age}\right]}^{-0.203}\times \left[0.742\;\mathrm{if}\;\mathrm{female}\right]\times 1.233, \end{array}$$

where serum creatinine is given as micromoles per liter and 1.233 is the adjusting coefficient. All laboratory assays were performed at the Department of Chemical Pathology, the Prince of Wales Hospital, Hong Kong, China using externally audited assays with precision within the manufacturers’ specifications.

Hong Kong is a cosmopolitan city of 7 million people with an ethnic majority of Southern Chinese. It has a heavily subsidized healthcare system that provides 95% of acute and chronic care. All public health enterprises are governed by the Hospital Authority and share a territory-wide Electronic Patient Record System including a death registry, which can be matched to a unique Hong Kong identity number held by all citizens. The system accurately captures prescription of all medications issued by public hospitals and clinics. Details of medication use, including the start and end dates of use, were retrieved from the system. We also used the Hospital Authority electronic system to identify first cancer events, fatal or non-fatal, using the International Classification of Diseases, Ninth Revision (code 140–208), censored on or before 30 July 2005.

### Statistical analyses

All analysis was performed using the Statistical Analysis System (Release 9.10, SAS Institute Inc., Cary, NC, USA). Separate logistic regression models were used to derive propensity scores for RAS inhibitors, statins, insulin, metformin, sulphonylurea and TZDs, using age, sex, use of alcohol and tobacco, diabetes duration, body mass index (BMI), systolic blood pressure (BP), LDL-C, HDL-C, triglyceride, log values of urinary ACR, eGFR, retinopathy, neuropathy, peripheral arterial disease, history of coronary heart disease, and stroke as independent variables.

We used time-fixed Cox regression models to evaluate the independent risk associations of cancer with use of RAS inhibitors, statins and blood glucose-lowering drugs (insulin, metformin, sulphonylurea, TZD) after excluding prevalent users and adjustment for propensity score. Using restricted cubic spline within Cox proportional hazard regression, we have previously demonstrated non-linear risk associations of BMI (<24.0 or ≥27.6 kg/m^2^), LDL-C, HDL-C and triglyceride with cancer in type 2 diabetes [10-13]. We identified additive interaction between low LDL-C and albuminuria on cancer risk [11,13]. We reported positive risk associations of cancer with LDL-C <2.8 mmol/L plus albuminuria, and with LDL-C ≥3.8 mmol/L [10,11,13]. Thus, in the final Cox model, the association of cancer and the use of specific drugs was adjusted by the following variables: BMI (<24.0 or ≥27.6 kg/m^2^), LDL-C-related risk indicators (LDL-C <2.8 mmol/L plus albuminuria, or LDL-C ≥3.8 mmol/L), non-linear associations of HDL-C and triglyceride with cancer, age, sex, occupation, use of alcohol and tobacco, duration of diabetes, systolic BP, HbA_1c_, and use of other drugs during follow-up (oral blood glucose-lowering drugs, insulin, lipid-lowering drugs and RAS inhibitors as appropriate). Of note, only cancer events that occurred after the commencement of the specific drug were captured in the exposed group. That is, if cancer developed prior to drug exposure, the event was considered to occur in the unexposed group. Lastly, we obtained the adjusted cumulative incidence of cancer stratified by a combination of attaining optimal glycemic control (HbA_1c_ <7%) at baseline and commencement of statins and RAS inhibitors on cancer risk after adjusting for all co-variables, using time-fixed Cox models (SPSS version 16, Chicago, IL, USA). Given 271 cancer events and 20 co-variables, over-fitting of the models was unlikely (the ratio of the number of endpoints to the number of co-variables was >10). All data were expressed as mean ± standard deviation (SD) or median (interquartile range (IQR)), or HR with 95% confidence intervals (CI). A *P*-value less than 0.05 (two-tailed) was considered significant.

### Addressing potential biases in pharmacoepidemiological analysis

In view of the controversies in pharmacoepidemiological analysis, there is a need to explain potential biases in analyzing the association of drug use with clinical outcomes in a non-clinical trial setting and the way to tackle these anticipated biases in this analysis. First, because the condition associated with drug use may contribute to the clinical outcome, for which the drug is prescribed to reduce risk, the propensity score for use of the drug is used to adjust for the drug indication [14]. Second, inclusion of existing users of drugs of interest will introduce prevalent user bias. Here, existing users at baseline may have an inherent advantage over non-users, being more likely to survive the initial event that prompted drug initiation and more tolerant of and/or better adhered to the drug of interest. Furthermore, alteration of risk profile by the drug in question complicates statistical adjustment of these risk factors. Exclusion of prevalent users by including only new users in the analysis is a common procedure to eliminate prevalent user bias. Third, and most controversial, is the exposure misclassification related to immortal time. Immortal time refers to the initial period of follow-up among drug users, when the outcome of interest cannot occur due to the definition of drug use. To control for immortal time bias, Suissa *et al.*[15] and Levesque *et al.*[16] suggested using either a time-dependent Cox model or, alternatively, the removal of the immortal time period from the user cohort in a time-fixed Cox model. We have previously demonstrated that using time-dependent drug-exposure Cox models to estimate the effects of drug use in diabetes results in a severely inflated HR [4], probably because of a failure to consider for parallel metabolic worsening in a time-dependent manner. In this present analysis, we re-examined the validity of the different statistical procedures in quantifying drug effect. To this end, we analyzed the association of statins use with cardiovascular events in the Hong Kong Diabetes Registry using various Cox models, and compared the results against the known effect sizes of statins on cardiovascular outcome from previous interventional trials.

### Validation of methods to control for immortal time bias

The benefit of statins in reducing cardiovascular disease is well proven based on randomized controlled trials. Using the Hong Kong Diabetes Registry, we compared the effect of statins use on incident cardiovascular events using the following statistical procedures: time-dependent drug-exposure Cox models with adjustment for co-variables at enrolment in non-users of statins and time-dependent co-variables at the time of use of the statins in users [15,16]; time-fixed Cox models with exclusion of immortal time among statins users, with adjustment for co-variables at enrolment in statins non-users and co-variables in statins users [17], that is, moving the start point of follow-up from enrolment to the end of immortal time for statins users; and time-fixed Cox model with inclusion of immortal time and adjustment for co-variables at enrolment for both users and non-users of statins.

To obtain time-dependent values of co-variables, we calculated partial regression coefficients of age (β_a_) and duration of diabetes (β_b_) for demographic and metabolic co-variables at enrolment in patients who were started statins during follow-up. Next, we estimated the values of BMI, systolic BP, HbA_1c_, LDL-C, HDL-C, triglyceride, urine ACR and eGFR at the time of commencing statins, using the following formula:

$$X_{t}=X_{b}+\beta_{a}T_{i}+\beta_{b}T_{i},$$

where X_t_ is the value at the time of starting statins during follow-up, X_b_ is the value at baseline, and T_i_ is the immortal time.

The validation showed that a time-fixed Cox model with inclusion of immortal time, that is, ignoring immortal time bias, produced a HR that was closest to the values reported in literature (Additional file 1: Table S1). The other models, particularly the time-dependent Cox model, tended to inflate the HR, even after adjustment for time-dependent co-variables and propensity score. Our findings strongly supported the use of a time-fixed Cox regression to estimate drug effects in our cohort. This method was used in all our analyses of drug use with cancer risk.

## Results

At enrolment, the median age of the cohort was 57 years (IQR: 47 to 67), duration of diabetes was 6 years (IQR: 2 to 11), and 46.1% were male (*n* = 2,808). Median follow-up was 4.9 years (IQR: 2.8 to 7.0), with a mean of 4.8 ± 2.4 years, during which 271 patients (4.4%) developed cancer with an incidence of 9.2 per 1,000 person-years (95% CI: 8.1, 10.3). Patients with incident cancers were older, more likely to smoke cigarettes and drink alcohol, had longer disease duration of diabetes, and had higher systolic BP and urinary ACR but lower eGFR and triglyceride at baseline than those without cancer. They were less likely to be started on lipid-lowering agents including statins and fibrates as well as oral blood glucose-lowering drugs including metformin and TZDs during follow-up (Table 1).

**Table 1:** Clinical and biochemical characteristics and use of medications of the study cohort stratified by cancer status

	**Patients without cancer**	**Patients with cancer**	
	**(*n*** **= 5,832)**	**(*n*** **= 271)**	
	**Median (IQR**^**a**^**) or % ( *n *)**	**Median (IQR**^**a**^**) or % ( *n *)**	***P***
**Baseline variables**			
Age (years)	57 (20)	66 (15)	<0.0001^b^
Male gender	45.8% (2,669)	51.3% (139)	0.0743^c^
Employment status			<0.0001^c^
Full-time	33.1% (1,928)	17.7% (48)	
Housewife	28.6% (1,665)	28.8% (78)	
Retired	27.9% (1,628)	46.5% (126)	
Others	10.5% (611)	7.0% (19)	
Smoking status			<0.0001^c^
Ex-smoker	15.10% (878)	18.7% (38)	
Current smoker	14.8% (862)	23.2% (47)	
Alcohol drinking status			<0.0001^c^
Ex-drinker	11.8% (688)	21.0% (51)	
Current drinker	7.3% (427)	7.4% (20)	
Body mass index (kg/m^2^)	24.8 (4.9)	24.3 (4.7)	0.1296^b^
Duration of diabetes (years)	6 (9)	8 (10)	0.0202^b^
Glycated hemoglobin HbA1c (%)	7.2 (2.0)	7.4 (2.3)	0.3586^b^
Systolic BP (mmHg)	134 (27)	137 (25)	0.0011^b^
Diastolic BP (mmHg)	75 (14)	75 (16)	0.6152^b^
Glycated hemoglobin (%)	7.2 (2.0)	7.4 (2.3)	0.3586^b^
LDL-C (mmol/L)	3.10 (1.23)	3.10 (1.30)	0.8872^b^
HDL-C (mmol/L)	1.26 (0.43)	1.25 (0.51)	0.3846^b^
Triglyceride (mmol/L)	1.34 (1.02)	1.23 (0.77)	0.0014^b^
Total cholesterol (mmol/L)	5.10 (1.33)	5.00 (1.42)	0.3083^b^
ACR (mg/mmol)	2.06 (10.47)	3.45 (14.18)	0.0023^b^
eGFR (ml/min/1.73m^2^)	103.1 (41.6)	98.8 (37.3)	0.0184^b^
Prior myocardial infarction	2.0% (114)	2.6% (7)	0.4683^d^
Prior stroke	4.5% (261)	3.7% (10)	0.5396^c^
Death (all-cause) during follow-up	6.3% (369)	50.2% (163)	<0.0001^c^
**Use of medications 2.5 years prior to enrolment**			
Statins	15.5% (903)	10.3% (28)	0.0211^c^
Fibrates	4.5% (261)	3.3% (9)	0.3664^c^
ACEIs/ARBs	29.5% (1,718)	28.8% (78)	0.8114^c^
Insulin	23.9% (1,394)	25.8% (70)	4676^c^
Metformin	56.6% (3,303)	52.4% (142)	0.1691^c^
Sulphonylurea	62.4% (3,640)	63.5% (172)	0.7260^c^
TZDs	0.5% (28)	0.4% (1)	0.7949^c^
**Use of medications during follow-up**^**e**^			
Statins	38.6% (2,249)	22.5% (61)	<0.0001^c^
Fibrates	10.2% (595)	5.9% (16)	0.0212^c^
ACEIs/ARBs	57.9% (3,378)	52.4% (142)	0.0720^c^
Insulin	37.7% (2197)	36.2% (98)	0.6161^c^
Metformin	74.5% (4,347)	63.8% (173)	<0.0001^c^
Sulphonylurea	71.3% (4,160)	69.4% (188)	0.4864^c^
TZDs	6.8% (398)	0.7% (2)	<0.0001^c^

^a^interquartile range, IQR, for variables with skewed distribution; ^b^derived from the Wilcoxon two-sample test; ^c^derived from Chi-squared test; ^d^derived from the Fisher’s exact test; ^e^including use started on or before enrolment but continued during follow-up period. ACEIs, angiotensin-converting enzyme inhibitors; ACR, spot urine albumin-to-creatinine ratio; ARBs, angiotensin II receptor blockers; BP, blood pressure; eGFR, estimated glomerular filtration rate; HDL-C, high-density lipoprotein cholesterol; IQR, interquartile range; LDL-C, low-density lipoprotein cholesterol; RAS, renin-angiotensin system; TZD, thiazolidinedione.

Table 2 shows the HR of different drugs for cancer risk after excluding prevalent users and adjusting for propensity score (model 1); co-variables including LDL-C-related risk indicators (that is, LDL-C <2.8 mmol/L plus albuminuria or LDL-C ≥3.8 mmol/L), non-linear associations of HDL-C and triglyceride with cancer, BMI (<24, ≥27.6 kg/m^2^), HbA_1c_, age, sex, occupation, use of alcohol and tobacco, disease duration of diabetes, and systolic BP; and drug use during follow-up without (model 2) and with propensity score for the drug in question (model 3).

**Table 2:** Hazard ratios of drug use for cancer in patients with type 2 diabetes after excluding prevalent users for the drug in question using a non-time-dependent Cox regression model, with adjustment for co-variables, propensity score and drug use during follow-up

**Users versus non-users**	**Sample size**	**Number of events**	**Hazard ratio (95% CI)**	***P***
ACEIs or ARBs				
Model 1	4,307	199	0.38 (0.27, 0.53)	<0.00001
Model 2	4,307	199	0.60 (0.43, 0.84)	0.0027
Model 3	4,307	199	0.55 (0.39, 0.78)	0.0009
Statins				
Model 1	5,172	243	0.36 (0.24, 0.53)	<0.0001
Model 2	5,172	243	0.47 (0.32, 0.70)	0.0002
Model 3	5,172	243	0.47 (0.31, 0.70)	0.0003
Insulin				
Model 1	4,639	201	0.48 (0.31, 0.73)	0.0006
Model 2	4,639	201	0.59 (0.39, 0.89)	0.0110
Model 3	4,639	201	0.58 (0.38, 0.89)	0.0119
Metformin				
Model 1	2,658	129	0.38 (0.25, 0.56)	<0.0001
Model 2	2,658	129	0.39 (0.25, 0.61)	<0.0001
Model 3	2,658	129	0.39 (0.25, 0.61)	<0.0001
Sulphonylurea				
Model 1	2,291	99	0.45 (0.29, 0.72)	0.0008
Model 2	2,291	99	0.44 (0.27, 0.73)	0.0013
Model 3	2,291	99	0.44 (0.27, 0.73)	0.0014
TZDs				
Model 1	6,074	270	0.12 (0.03, 0.50)	0.0033
Model 2	6,074	270	0.17 (0.04, 0.69)	0.0133
Model 3	6,074	270	0.18 (0.04, 0.72)	0.0153

Model 1 adjusted for propensity scores (c-statistics = 0.80 for ACEIs or ARBs; 0.80 for statins; 0.79 for insulin; 0.73 for metformin; 0.66 for sulphonylurea; and 0.74 for TZDs), which were calculated using logistic regression procedures with age, sex, body mass index, low- and high-density lipoprotein cholesterols (LDL-C and HDL-C), triglyceride, use of tobacco and alcohol, HbA_1c_, systolic blood pressure, natural log of the albumin-to-creatinine ratio, estimated glomerular filtration rate, duration of disease, peripheral arterial disease, retinopathy, neuropathy, prior myocardial infarction and stroke as independent variables. Model 2 adjusted for LDL-C-related risk indicators (that is, LDL-C <2.8 mmol/L plus albuminuria or LDL-C ≥3.8 mmol/L); non-linear associations of HDL-C and triglyceride for cancer; body mass index (<24, ≥27.6 kg/m^2^); age; sex; employment status; use of tobacco and alcohol; duration of disease; systolic blood pressure; and use of statins, ACEIs/ARBs, fibrates, sulphonylurea, metformin and TZDs during follow-up. Model 3 adjusted for all factors in model 2 plus the propensity score of the drug in question. No patient had been exposed to the drug in question for at least 2.5 years prior to enrolment. ACEIs, angiotensin-converting enzyme inhibitors; ARBs, angiotensin II receptor blockers; CI, confidence interval; TZD, thiazolidinediones.

**Figure 2:**
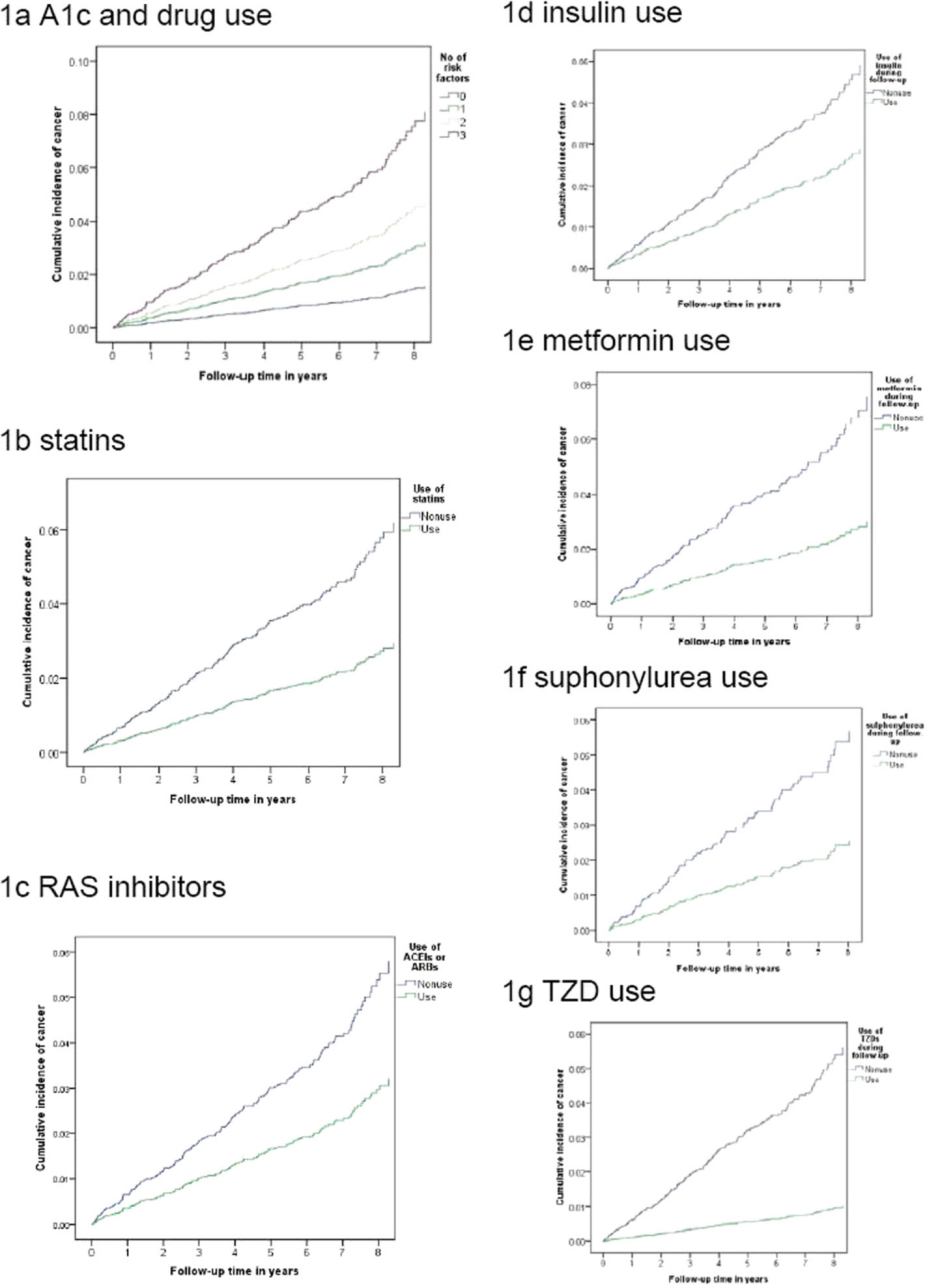
**Cumulative incidence of all-site cancer in patients with type 2 diabetes.** Stratified by drug usage and attainment of glycemic goal after adjustment for LDL-C-related risk indicators (that is, LDL-C < 2.8 mmol/L plus albuminuria or LDL-C ≥3.8 mmol/L), non-linear associations of HDL-C and triglyceride with cancer, BMI (<24, ≥27.6 kg/m^2^), HbA_1c_, (except for Figure 1a) age, sex, employment status, use of alcohol and tobacco, duration of disease, and systolic blood pressure**.** Additional adjustments were made for propensity scores of the drug in question and use of other medications during follow-up as appropriate. All analyses were performed after excluding prevalent drug users. Abbreviation list: LDL-C, low-density lipoprotein cholesterol; HDL-C, high-density lipoprotein cholesterol; BMI, body mass index; HbA_1c_, glycated haemoglobin; RAS, renin-angiotensin system; TZD, thiazolidinedione.

Use of statins, RAS inhibitors, insulin, metformin, sulphonylurea and TZD was independently associated with a 40% to 60% risk reduction for all-site cancer. Figure 2 shows the cumulative incidence of cancer in patients stratified by the number of risk factors defined as HbA_1c_ ≥7% and non-use of statins and/or RAS inhibitors, as well as that by usage of individual drugs, adjusted for all confounders. The numbers of patients in different risk groups during the follow-up period with reference to Figure 1 are shown in Additional file 1: Table S2. Compared with patients with HbA_1c_ < 7% who started on statins and RAS inhibitors, those with high HbA_1c_ and treated with neither drug had a four-fold increased risk of incident all-site cancer (incidence per 1,000 person-years (HR, (95% CI)): no risk factor, 3.40 (0.07, 6.72); one risk factor, 6.34 (4.19, 8.50); two risk factors, 8.40 (6.60, 10.20); three risk factors, 13.08 (9.82, 16.34); *P* <0.001). We repeated the analyses by specific cancer site but the number of cancers at specific sites was too small to yield any significant results. Distribution of cancer sites among the patients who developed cancers (*n* = 271) and some patients had developed cancers at more than one site were shown in table S3. (Additional file 1: Table S3).

## Discussion

In this prospective cohort of Chinese patients with type 2 diabetes without prior history of cancer or exposure to the drugs in question, suboptimal glycemic control (HbA_1c_ ≥7%) and non-use of RAS inhibitors and statins were associated with increased cancer risk in an additive manner. While the incidence of cancer was 9.21 per 1,000 person-years in the entire cohort, non-users of RAS inhibitors and statins with HbA_1c_ ≥7% had four-fold higher incidence of cancer (13.08 per 1,000 person-years) than users of both drugs with HbA_1c_ <7% (3.40 per 1,000 person-years). Consistently, treatment with insulin, metformin, sulphonylurea and TZD was associated with 40% to 80% reduced cancer risk after adjusting for co-variables, drug indications, use of other drugs, and lipid-associated risk factors for cancer.

### Effects of hyperglycemia on cancer risk

Warburg first reported in the 1920s that, under anaerobic conditions, respiration due to fermentation (insufficient oxygen) favored cancer cell growth over normal cell growth, which is more dependent on aerobic respiration (sufficient oxygen) [18]. Diabetes is a disorder of energy metabolism caused by inadequate insulin action, either quantitatively or qualitatively. The use of fat as an alternative energy substrate in diabetes promotes free fatty acid production and oxidative stress. The latter can be perpetuated by generalized vasculopathy with insufficient oxygen and glucose delivery at a tissue level. Hyperglycemia can also activate cellular signals such as angiotensin II, nicotinamide adenine dinucleotide phosphate oxidase, aldose reductase, protein kinase C, advanced glycation end products and nuclear factor kappa B, which interact to cause abnormal cell cycles through oxidative stress and inflammation [3,19]. Other researchers have reported the proliferative effects of hyperglycemia on pancreatic cancer cells [20] through dysregulation of multiple growth-promoting pathways.

In support of these mechanistic studies, we [21] and others [1] have reported the near-linear relationships of fasting plasma glucose and HbA_1c_ with cancer risk. For every 1% increase in HbA_1c_, there is an 18% increase in cancer risk in patients with type 2 diabetes [21]. Other researchers have reported risk association between cancer and fasting plasma glucose independent of obesity [22] and between pancreatic cancer and 2-hour post-load plasma glucose [23] in non-diabetic subjects. Similarly, patients with cancer in the highest tertile of fasting plasma glucose have the lowest survival rate compared to those in the lower tertiles [24].

### Drug usage and cancer risk

Despite the controversies regarding the use of insulin and cancer risk, in a randomized clinical trial, the ORIGIN (Outcomes Reduction with an Initial Glargine Intervention) study, early treatment with insulin glargine in patients with dysglycemia, including pre-diabetes and type 2 diabetes, was not associated with increased cancer risk [25]. In this randomized trial, the cancer incidence was 1.32 per 100 person-years, similar to that of 0.92 per 100 person-years in the present report. In a previous analysis using validated methods (that is, a sensitivity analysis with use of the time-fixed Cox model), insulin users had a 51% risk reduction in cancer risk compared with non-users, probably due to insulin’s effects on blood glucose lowering [21].

Both diabetes and cancer are complex diseases due to multi-causality [26]. There are many reasons for the inconsistent, and sometimes conflicting, results regarding the risk associations of cancer with drugs from studies that were often not designed to address the question in the first place. These include heterogeneity of study populations, quality of data collection, inclusion and exclusion of various confounders, and study design and setting. Recently, we summarized potential biases in pharmacoepidemiological analysis including lack of adjustment for confounders and drug indication (for example, using the propensity score), inclusion of prevalent drug users, adjustment for immortal time and use of time-dependent analysis [27]. In our present validation of various statistical methods on estimating drug effects, we demonstrated that different conclusions can be drawn regarding the risk and benefit of statins on cardiovascular diseases using the same database. Not to disregard the theoretical possibility of immortal time bias, our results clearly suggest that attempts to remove immortal time using alternative methods such as time-dependent Cox regression were prone to biases from other sources, as evidenced by the generation of HRs that were severely inflated. Conversely, the use of conservative time-fixed Cox regression with inclusion of immortal time produced an effect size most comparable to that obtained in major interventional trials, suggesting that immortal time may have only limited impact in pharmacoepidemiological analysis.

### Insulin deficiency versus insulin resistance

Hyperglycemia can dysregulate intracellular signaling pathways and may impact on cancer cell growth [3,5]. Both insulin deficiency and resistance can contribute to hyperglycemia, which can vary between individuals and within the same individual over time. Chronic hyperinsulinemia increases risk of cancers of the colon, endometrium and probably other sites, including pancreas and kidneys [28]. Using chemically induced hepatocarcinogenesis in diabetic model mice genetically deficient for insulin, volumes of hepatocellular tumors were more than two-fold larger in the insulin-deficient mice compared with the normal controls, suggesting that insulin-independent mechanisms might operate in liver tumor growth [29].

As such, we and others have also reported the attenuated cancer risk associated with metformin or TZD treatment. Both ApoA-1 and metformin interact with tumor-suppressive liver kinase B1 to activate adenosine monophosphate-activated protein kinase. In this context, metformin-treated type 2 diabetic patients with low HDL-C had markedly attenuated cancer risk than non-users, suggesting possible drug-sub-phenotype interaction. In another sub-analysis, we reported 80% risk reduction in cancer in patients with type 2 diabetes treated with TZD, probably due to TZD’s ameliorating effects on gluco-lipotoxicity, inflammation and oxidative stress through fat redistribution [2]. In a national database from Taiwan, patients with diabetes treated with any anti-diabetic drugs had 10% to 60% lower risk of liver cancer than non-users, with metformin and TZD having the largest effect size [30]. In a prospective cohort of patients with breast cancers, users of TZD and metformin had better survival rates than non-users, with both drugs conferring 50% risk reduction in mortality [31]. In a recent meta-analysis, the risk association of TZD with bladder cancer appeared to be limited to pioglitazone, suggesting a rare drug-specific, rather than class effect [32].

### Inhibition of HMGCR and RAS pathways on cancer risk

Although there are strong experimental and clinical data supporting the anti-cancer effects of statins [33], the situation is more controversial with respect to RAS inhibitors. In response to a report on risk association of cancer with RAS inhibitors, we used data from our registry to point out the independent risk association of cancer with propensity score for RAS inhibitors. Our findings suggest that microenvironments associated with RAS activation linked to hyperglycemia, high BP and renal dysfunction might promote cancer growth in predisposed subjects [4]. Herein, experimental studies suggest intimate relationships between insulin, sterol regulatory element-binding proteins (SREBPs), insulin-like growth factor-1 (IGF1) and 3-hydroxy-3-methyl-glutaryl-coenzyme-A-reductase (HMGCR) pathways (Figure 2). Insulin regulates triglyceride synthesis through activation of the SREBP-1c pathway while IGF1 activates the SREBP-1a pathway to promote cholesterol synthesis via the HMGCR pathway. However, the latter can lead to increased production of mevalonate and Ras signals, which are known oncogenes. Since both insulin and IGF1 pathways share cognate receptors, insufficient insulin action may cause dysregulation of the IGF1-SREBP-HMGCR-mevalonate-Ras pathway to increase cancer risk [2]. In a uninephrectomized rat model characterized by sequential development of dysglycemia, renal dysfunction and renal cancer, we found activation of the RAS pathway, reduced expression of IGF-binding proteins and increased expression of HMGCR, protein kinase C ξ and Akt (or protein kinase B) which were reversed by treatment with RAS inhibitors, with reduced cancer growth, suggesting crosstalk between the RAS and HMGCR pathways [34].

### Strengths and weaknesses

Despite the limitations of observational studies and pharmacoepidemiological analysis, there is now a growing body of clinical and experimental data that support the cancer-promoting effects of hyperglycemia and, importantly, the potentially preventable nature of cancer. In this article, we have used detailed analysis with corroborative evidence from independent studies to argue for the possible causal role of hyperglycemia and associated changes in microenvironment in promoting cancer growth using the criteria set out by Bradford-Hill [35]. The latter include strength of associations (cancer risk and diabetes with odds ratio ≥1.3 to 3) [1], specificity (cancer risk attenuated only by adjusting blood glucose) [1], temporal relationship (prospective cohorts and association of cancer risk in pre-diabetes) [22,36], biological gradient (linear relationship of cancer risk with HbA_1c_ and plasma glucose) [1,21], biological plausibility (activation of multiple and interlinked pathways by hyperglycemia) [3], coherence (high prevalence of diabetes in cancer and improved cancer survival in patients treated with blood glucose lowering drugs), experiments [29] and analogy (growth-promoting effects of hyperglycemia in renal and endothelial cells) [37]. Of note, socioeconomic status such as educational attainment, income, employment and occupation might lead to preferential prescription of certain drugs, which might potentially lead to allocation bias. In this regard, we had adjusted for employment status and the difference before and after the adjustment had little impacts on the effect sizes of drug use reported in this study. Since Hong Kong has a heavily subsidized healthcare system and all patients only have to pay a nominal fee of less than 2 US dollars per drug item lasting for 3 to 4 months with waiving of all fees if they are on social security, socioeconomic status or education level were not likely to be important factors in drug selection in the present cohort. We were not sure about the effects of these socioeconomic factors on drug compliance but this was likely to be random and equally distributed in both groups. That said, due to the public nature of the hospital, there might be an under-representation of patients from the younger population and upper socioeconomic class who might seek additional anti-cancer treatment in the private sector. Due to the low numbers of cancers at specific sites, we could not explore the associations between diabetes and site-specific cancer.

Taken together, the systematic and comprehensive documentation of baseline risk factors, long duration of follow-up, concurrent drug use, and clinical outcomes are the major strengths of this cohort. We cannot exclude inclusion of subclinical cases of cancer, although only 39.1% (*n* = 106) of cancer cases occurred within the first 2 years of enrolment. We cannot exclude other unmeasured variables (for example, better treatment compliance, lifestyle factors, quality of care) associated with better glycemic control and use of these drugs, which might confound these associations. Despite adjustment for propensity score, potential confounding by indication remains.

## Conclusion

Despite the proven benefits of control of multiple risk factors in type 2 diabetes, in real practice, glycemic control is often suboptimal with omission of many life-saving drugs. In developing areas such as Asia where beta cell insufficiency, metabolic syndrome, low grade chronic infections (for example, hepatitis B), early onset of disease and renal dysfunction characterized by oxidative stress and microinflammation are highly prevalent [6], our findings carry important public and personal health implications. In this light, Asians living in the US had higher rates of gastric cancer than their white counterparts [38]. Against this background, our findings suggest that use of RAS inhibitors and statins may prevent the adverse consequences of abnormal microenvironment in diabetes that may promote loss of functions or abnormal cell growth. While well-designed prospective cohorts, randomized trials and mechanistic studies are needed to confirm our findings, given the complex yet probabilistic nature of the effects on host-environment-lifestyle interactions on clinical outcomes, our data also highlight the opportunities for preventing multiple morbidities in diabetes by strengthening our healthcare system to optimize risk factor control and promote patient empowerment with ongoing monitoring and evaluation [39].

## Abbreviations

ACR: albumin-to-creatinine ratio; BMI: body mass index; BP: blood pressure; CI: confidence interval; eGFR: estimated glomerular filtration rate; HDL-C: high-density lipoprotein cholesterol; HMGCR: 3-hydroxy-3-methyl-glutaryl-coenzyme-A-reductase; HR: hazard ratio; IGF1: insulin-like growth factor-1; IQR: interquartile range; LDL-C: low-density lipoprotein cholesterol; RAS: renin-angiotensin system; SD: standard deviation; SREBPs: sterol regulatory element-binding proteins; TZD: thiazolidinedione.

## Competing interests

JC is a board member of the Asia Diabetes Foundation. She is a consultant for AstraZeneca, Bristol-Myers Squibb, Daiichi-Sankyo, GlaxoSmithKline, Merck Sharp & Dohme, Pfizer, Sanofi-Aventis and Qualigenics. She has received honoraria, travel expenses, and/or payments for development of educational presentations from AstraZeneca, Bayer, Bristol-Myers Squibb, Daiichi-Sankyo, Eli Lilly, GlaxoSmithKline, Merck Serono, Merck Sharp & Dohme, Nestle Nutrition Institute, Novo Nordisk, Pfizer, Roche, Sanofi and Takeda. RM has received honorarium for consultancy or giving lectures from AstraZeneca, Pfizer and Sanofi. AK has received honorarium for consultancy or giving lectures from Nestle Nutrition Institute, Merck Serono, Pfizer, Eli Lilly, Roche, Sanofi, Jassen and AstraZeneca. All other authors declare that they have no competing interests.

## Authors’ contributions

AK, XY, WS, HL, KC, GX and JC researched data. AK, XY and JC wrote the manuscript. AK, WS, RM, AL, RO, LY, CCC and JC collected data. All authors read and approved the final manuscript.

## Pre-publication history

The pre-publication history for this paper can be accessed here:

http://www.biomedcentral.com/1741-7015/12/76/prepub

## Supplementary Material

Additional file 1: Table S1 Validation of methods to control for immortal time bias: HRs of the use of statins during follow-up for the risk of cardiovascular disease in 4,657 patients with type 2 diabetes and non-use of statins in 2.5 years prior to enrolment. **Table S2.** Numbers of patients in different risk groups during the follow-up period with reference to Figure 1. **Table S3.** Distribution of cancer sites among 271 subjects who had developed cancers [40]. Click here for file
