# Supplementary material for: Additive effects of blood glucose lowering drugs, statins and renin-angiotensin system blockers on all-site cancer risk in patients with type 2 diabetes
Source: BMC Med. 2014 May 13;12:76. doi: 10.1186/1741-7015-12-76 (PMC4046510; doi:10.1186/1741-7015-12-76)
Supplement: Additional file 1: Table S1 — Validation of methods to control for immortal time bias: HRs of the use of statins during follow-up for the risk of cardiovascular disease in 4,657 patients with type 2 diabetes and non-use of statins in 2.5 years prior to enrolment. Table S2. Numbers of patients in different risk groups during the follow-up period with reference to Figure 1. Table S3. Distribution of cancer sites among 271 subjects who had developed cancers [40]. [file 1741-7015-12-76-S1.doc]

**Supplementary Table 1.** **Validation of methods to control for immortal time bias:** Hazard ratios of the use of statins during follow-up for the risk of cardiovascular disease (CVD) in 4,657 patients with type 2 diabetes and nonuse of statins in 2.5 years prior to enrolment

| Use vs. nonuse of statins | Hazard ratio | 95% CI | P value | Inflation§ |
| --- | --- | --- | --- | --- |
| **Time-fixed Cox model with inclusion of immortal time** |  |  |  |  |
| Univariable analysis | 0.90 | 0.71-1.14 | 0.3663 | 42.86% |
| Adjusted for covariables at enrolment† | 0.55 | 0.42-0.72 | <0.0001 | −12.70% |
| Further adjusted for drug use‡ | 0.64 | 0.48-0.84 | 0.0012 | 1.59% |
| Further adjusted for propensity score for statins¶ | 0.65 | 0.50-0.86 | 0.0024 | 3.17% |
| **Time-dependent Cox model with inclusion of immortal time** |  |  |  |  |
| Univariable analysis | 2.00 | 1.57-2.56 | <0.0001 | 217.46% |
| Adjusted for covariables at enrolment | 1.37 | 1.04-1.81 | 0.0239 | 117.46% |
| Adjusted for covariables at enrolment among non-statin users or time-dependent covariables in users†† | 1.19 | 0.91-1.57 | 0.2118 | 88.89% |
| Further adjusted for drug use‡ | 1.33 | 1.01-1.76 | 0.0444 | 111.11% |
| Further adjusted for propensity score for statins¶¶ | 1.34 | 1.01-1.78 | 0.0463 | 112.70% |
| **Time-fixed Cox model with exclusion of immortal time among statin users** |  |  |  |  |
| Univariable analysis | 1.65 | 1.29-2.10 | <0.0001 | 161.90% |
| Adjusted for covariables at enrolment | 1.02 | 0.77-1.34 | 0.9148 | 61.90% |
| Adjusted for covariables at enrolment in non-statins users or at the time of use of statins in statins users†† | 0.88 | 0.67-1.17 | 0.3811 | 39.68% |
| Further adjusted for drug use‡ | 0.96 | 0.72-1.27 | 0.7621 | 52.38% |
| Further adjusted for propensity score for statins¶¶ | 1.00 | 0.75-1.33 | 0.9978 | 58.73% |

Abbreviations: ACEIs, angiotensin-converting enzyme inhibitors; ARBs, angiotensin II receptor blockers; LDL-C, low-density lipoprotein cholesterol; BMI, body mass index; SBP, systolic blood pressure; ACR, urinary albumin to creatinine ratio; eGFR, estimated glomerular filtration rate.

†Covariables at enrollment included age, sex, duration of diabetes, BMI, smoking status, alcohol use, LDL-C, high density-lipoprotein cholesterol, triglyceride, SBP, HbA_1c_ and Ln (ACR+1) and eGFR;

‡ Drug use included use of non-ACEI/ARB antihypertensive drugs at enrollmemt and use of insulin, ACEIs/ARBs, gliclazide, glibenclamide and thiazolidinediones from enrollment (or initiation of statin therapy in time-fixed models with exclusion of immortal time) to CVD, death or 30 July 2005 whichever came first;

¶Propensity score of use of statins during follow-up were estimated from age, sex, LDL-C, triglyceride, HbA_1c_, SBP, Ln(ACR+1) and eGFR at enrollment that were selected by stepwise selection (P<0.30 for entry and stay)(the c-statistic=0.79);

¶¶Propensity score of use of statins during follow-up were estimated from age, LDL-C, triglyceride, HbA_1c_, SBP, Ln(ACR+1) and eGFR that were selected by stepwise selection (P<0.30 for entry and stay) (the c-statistic=0.81); The values of covariables at enrollment in non-statin users or estimated values of these covariables at the time of use of statins in users were used in the calculation;

††Covariables at the time of initiation of statin therapy during follow-up among statin users were estimated using partial coefficients of age and duration of diabetes that were obtained from all other covariables at enrollment, using the formula: X_t_=X_b_+β_a_ T_i_ +β_b_ T_i_, where X_t_ is the value at the time of use of statins during follow-up, X_b_ is the value at baseline, and T_i_ is the immortal time.

§Inflation percentage was calculated based on the effect size, 0.63, from Colhoun et al. (45).

**Supplementary Table 2:** Numbers of patients in different risk groups during the follow up period with reference to Figure 1.

|  | 0 | 1 | 2 | 3 | 4 | 5 | 6 | 7 | 8 |
| --- | --- | --- | --- | --- | --- | --- | --- | --- | --- |
| No of risk factors |  |  |  |  |  |  |  |  |  |
| 0 | 189 | 188 | 178 | 167 | 153 | 144 | 127 | 92 | 31 |
| 1 | 909 | 890 | 844 | 773 | 705 | 596 | 486 | 322 | 124 |
| 2 | 1872 | 1803 | 1629 | 1438 | 1280 | 1081 | 862 | 605 | 238 |
| 3 | 926 | 869 | 792 | 701 | 616 | 492 | 373 | 257 | 113 |
| Use of ACEIs or ARBs |  |  |  |  |  |  |  |  |  |
| No | 2582 | 2424 | 2131 | 1854 | 1611 | 1315 | 1052 | 710 | 271 |
| Yes | 3404 | 3231 | 2874 | 2499 | 2109 | 1664 | 1239 | 796 | 274 |
|  |  |  |  |  |  |  |  |  |  |
| Use of insulin |  |  |  |  |  |  |  |  |  |
| No | 3666 | 3416 | 2972 | 2537 | 2156 | 1746 | 1390 | 951 | 372 |
| Yes | 973 | 959 | 906 | 825 | 792 | 614 | 488 | 353 | 129 |
|  |  |  |  |  |  |  |  |  |  |
| Use of metformin |  |  |  |  |  |  |  |  |  |
| No | 1107 | 1012 | 870 | 722 | 593 | 454 | 375 | 272 | 99 |
| Yes | 3532 | 3363 | 3008 | 2640 | 2292 | 1906 | 1503 | 1032 | 402 |
|  |  |  |  |  |  |  |  |  |  |
| Use of statins |  |  |  |  |  |  |  |  |  |
| No | 3757 | 3542 | 3138 | 2273 | 2369 | 1952 | 1510 | 1012 | 376 |
| Yes | 1415 | 1380 | 1284 | 1152 | 1004 | 808 | 609 | 408 | 155 |
|  |  |  |  |  |  |  |  |  |  |
| Use of sulphonylurea |  |  |  |  |  |  |  |  |  |
| No | 1472 | 1375 | 1186 | 1001 | 848 | 623 | 489 | 310 | 115 |
| Yes | 819 | 806 | 769 | 726 | 656 | 584 | 493 | 370 | 151 |
|  |  |  |  |  |  |  |  |  |  |
| Use of TZDs |  |  |  |  |  |  |  |  |  |
| No | 5698 | 5362 | 4709 | 4071 | 3468 | 2768 | 2127 | 1407 | 507 |
| Yes | 376 | 370 | 355 | 329 | 285 | 222 | 165 | 99 | 38 |

Abbreviations: ACEIs, angiotensin-converting enzyme inhibitors; ARBs, angiotensin II receptor blockers; TZD, thiazolidinedione.

**Supplementary Table 3.** Distribution of cancer sitse among 271 subjects who had developed cancers.

| Cancer | Number of subjects* | % |
| --- | --- | --- |
| Any sites | 271 | 100% |
| Lip, oral cavity, and pharynx | 9 | 3.2% |
| Digestive organs and peritoneum | 132 | 48.7% |
| Esophagus and stomach | 22 | 8.1 |
| Colon and rectum rectosigmoid junction and anus | 51 | 18.8% |
| Liver and intrahepatic bile ducts | 39 | 14.4% |
| Respiratory and intrathoracic organs | 33 | 12.2% |
| Bone, connective tissue, skin and breast | 40 | 14.8% |
| Female breast | 24 | 8.9% |
| Genitourinary organs | 44 | 16.2% |
| Prostate | 14 | 5.2% |
| Lymphatic and hematopoietic tissue | 36 | 13.3% |
| Other and unspecified sites | 11 | 4.1% |

* Please note that some patients had cancers at more than one site, so the sum of the number of cancers was greater than the total number of subjects.
